# Optimizing the method for generation of integration-free induced pluripotent stem cells from human peripheral blood

**DOI:** 10.1186/s13287-018-0908-z

**Published:** 2018-06-15

**Authors:** Haihui Gu, Xia Huang, Jing Xu, Lili Song, Shuping Liu, Xiao-bing Zhang, Weiping Yuan, Yanxin Li

**Affiliations:** 1 State Key Laboratory of Experimental Hematology, Institute of Hematology and Blood Diseases Hospital, Center for Stem Cell Medicine, Chinese Academy of Medical Sciences and Peking Union Medical College, Tianjin, 200093 China; 2 Department of Transfusion Medicine, Shanghai Changhai Hospital, Second Military Medical University, 168 Changhai Road, Shanghai, 200433 China; 3 Key Laboratory of Pediatric Hematology and Oncology, Ministry of Health, Pediatric Translational Medicine Institute, Shanghai Children’s Medical Center, School of Medicine, Shanghai Jiao Tong University, Shanghai, 200127 China

**Keywords:** Induced pluripotent stem cell, Reprogramming, Integration free, Episomal vector

## Abstract

**Background:**

Generation of induced pluripotent stem cells (iPSCs) from human peripheral blood provides a convenient and low-invasive way to obtain patient-specific iPSCs. The episomal vector is one of the best approaches for reprogramming somatic cells to pluripotent status because of its simplicity and affordability. However, the efficiency of episomal vector reprogramming of adult peripheral blood cells is relatively low compared with cord blood and bone marrow cells.

**Methods:**

In the present study, integration-free human iPSCs derived from peripheral blood were established via episomal technology. We optimized mononuclear cell isolation and cultivation, episomal vector promoters, and a combination of transcriptional factors to improve reprogramming efficiency.

**Results:**

Here, we improved the generation efficiency of integration-free iPSCs from human peripheral blood mononuclear cells by optimizing the method of isolating mononuclear cells from peripheral blood, by modifying the integration of culture medium, and by adjusting the duration of culture time and the combination of different episomal vectors.

**Conclusions:**

With this optimized protocol, a valuable asset for banking patient-specific iPSCs has been established.

**Electronic supplementary material:**

The online version of this article (10.1186/s13287-018-0908-z) contains supplementary material, which is available to authorized users.

## Background

Induced pluripotent stem cells (iPSCs) are a type of pluripotent stem cell resembling embryonic stem cells (ESCs) that can be directly generated from somatic cells by transcription factors [1]. The first common source for human iPSC derivation was skin dermal fibroblasts [2]. Since that discovery, a variety of somatic tissue cells have been reprogrammed to pluripotency [3–5]. Mononuclear cells (MNCs) from peripheral blood (PB) have been widely accepted as a more convenient and almost unlimited source of cells for reprogramming [6–10].

The original method using retroviral or lentiviral vectors expressing Oct4 (officially known as Pou5f1), Sox2, Klf4, and c-Myc (known as Myc) has high reprogramming efficiency, but neither type of viral vector (retroviral and lentiviral) is ideal for clinical application. Viral vectors carry a risk for insertion mutation, which can result in tumorigenicity and genomic instability of iPSCs [11]. To make iPSC-based therapies safer, great efforts have been exerted to establish the cells without integration of an exogenous sequence into the cellular genomes. These techniques include using recombinant proteins or mRNA as an alternative to exogenous DNA [12, 13], Sendai virus methods [14], and episomal methods [15, 16]. Although episomal vectors are the most practical and efficient of these options, the reprogramming efficiency of this strategy needs to be improved [17, 18].

In this study, we optimized isolation of MNCs, modified the supplementation of culture medium, and adjusted the duration of culture time and the combination of different episomal vectors to improve the reprogramming technology of using human PB cells as donor cells. These optimizations have important implications for the clinical applications of iPSCs.

## Methods

### Cell culture

Primary murine embryonic fibroblasts (MEFs) were obtained from 13.5-day CD-1 IGS mouse embryos and cultured in standard DMEM containing 10% FBS (Hyclone, Logan, UT, USA) and 2 mM l-glutamine. The MEF cells (passage 3) were irradiated at 60 Gy and then plated on gelatinized plates. Irradiated MEFs (2 × 10^5^ cells) were coated onto six-well plates to support the culture for iPSC generation.

iPSCs were usually maintained in a feeder-free culture system. Briefly, we precoated the well plates with Matrigel (BD Biosciences), and then seeded the iPSCs and cultured them with E8 medium. When iPSCs reached 30–60% confluence, they could be passaged routinely with EDTA (0.5 M/L).

### Isolation and preparation of MNCs from peripheral blood

All human whole blood samples were obtained from volunteers at the Institute of Hematology and Blood Diseases Hospital. Each PB sample was divided equally into four parts. MNCs were isolated from part 1 using standard Ficoll procedures by loading 35 ml of diluted blood (blood:PBS = 1:2) onto a 15-ml layer of Ficoll-Paque PREMIUM (*p* = 1.077 g/ml; Sigma Aldrich) in a 50-ml conical tube. MNCs were isolated from part 2 using red cell lysis buffer procedures, with the addition of 4-fold of ACK buffer to the blood and centrifugation after 20 min of incubation in a 37 °C water bath. Hydroxyethyl starch (HES; Sigma Aldrich) of 60-kDa molecular mass was added to PB from parts 3 and 4 in a 1:5 ratio. The supernatants collected after the sample remained stationary for 40 min at room temperature and were processed to yield MNCs either by the Ficoll method for part 3 or by the ACK method for part 4. In total, we used two isolating methods for enrichment of PB MNCs, including Ficoll and HES-Ficoll, and two isolating methods without enrichment, including ACK and HES-ACK. Except for MNCs, the other types of white blood cells would die quickly after being cultured in vitro, so we designated the isolated cells PB MNCs.

### Culture and expansion of MNCs from peripheral blood

PB MNCs were expanded for 4–10 days in a serum-free medium supplemented with a mixture of cytokines. The two main culture media (erythroid culture medium (ECM) and granulocyte culture medium (GCM)) were tested. ECM included IMDM (50%; Invitrogen) and Ham’s F12 (50%; Invitrogen) with ITS-X (100×; Invitrogen), chemically defined lipid concentrate (100×; Invitrogen), l-glutamine (100×; Invitrogen), ascorbic acid (0.05 mg/ml; Sigma), BSA (5 mg/ml; Sigma), l-thioglycerol (200 μM; Sigma), SCF (100 ng/ml; PeproTech), IL-3 (10 ng/ml; PeproTech), erythropoietin (2 U/ml; PeproTech), IGF-1 (40 ng/ml; PeproTech), dexamethasone (1 μM; Sigma), and holo-transferrin (100 μg/ml; R&D). GCM was supplemented with IMDM (50%) and Ham’s F12 (50%), ITS-X (100×), chemically defined lipid concentrate (100×), l-glutamine (100×), ascorbic acid (0.05 mg/ml), BSA (5 mg/ml), 1-thioglycerol (200 μM), Thrombopoietin (100 ng/ml; PeproTech), SCF (100 ng/ml), Flt3 ligand (100 ng/ml; PeproTech), granulocyte-colony stimulating factor (G-CSF) (100 ng/ml; PeproTech), and IL-3 (10 ng/ml). During culture, we quantified the living cells by FACS staining and counted them automatically using a cell number counting machine (Bio-Rad).

### Nucleofection and generation of iPSCs

Episomal vectors included three sets according to the different promoters: CAG, EF1, and SFFV. The CAG set included pEV CAG-OCT4-E2A-SOX2 (CAG-OS) and pEV CAG-MYC-E2A-KLF4 (CAG-MK); the EF1 set included pEV EF1-OCT4-E2A-SOX2 (EF1-OS) and pEV EF1-MYC-E2A-KLF4 (EF1-MK); and the SFFV set included pEV SFFV-OCT4-E2A-SOX2 (OS) and pEV SFFV-MYC-E2A-KLF4 (MK). To improve the reprogramming efficiency, we cloned pEV SFFV-BCL-XL (Bcl-XL), pEV SFFV-BCL2 (B), and pEV SFFV-Shp53 (Shp53). We added plasmids (4 μg OS (CAG-OS or EF1-OS), 4 μg MK (CAG-MK or EF1-MK) and 2 μg B (Shp53 or BCL-XL)) to a sterile Eppendorf tube and mixed them with 100 μl nucleofection buffer (Nucleofector™ Kits for Human CD34^+^ Cells, Cat. No. VPA-1003; Lonza), and then transferred the mix to the cell pellet (1 × 10^6^ cells). Using the kit-provided plastic pipette, we transferred the mixture of plasmids and cells into the provided cuvette to run the program (U008) on the nucleofection (2B; Lonza). After nucleofection, we directly transferred the mixture to the culture plate, which was already preseeded with feeder cells. The cells were cultured in reprogramming medium, which was composed of knockout DMEM/F12 medium (Invitrogen) and supplemented with 1% l-glutamine (Invitrogen), 2 mM nonessential amino acids (Invitrogen), 1% penicillin/streptomycin (Cat. No. G255; Invitrogen), 50 ng/ml FGF2 (Invitrogen), 1% ITS (BD Biosciences), and 50 μg/ml ascorbic acid (Sigma) for 7 days. The cells were then cultured in E8 medium (Invitrogen) until iPSCs were generated.

### Alkaline phosphate staining and immunocytochemistry

Alkaline phosphatase (AP) staining was performed using a Fast Red substrate kit (Invitrogen). For detection of pluripotent stem cell marker antigens, cells were fixed with PBS containing 4% paraformaldehyde for 10 min at room temperature. After being washed with PBS, the cells were incubated in PBS containing 0.1% Triton X-100 for 20 min at room temperature. Fixed cells were stained with the primary antibodies SSEA-4 (1:100; Stemgent), TRA-1-60 (1/200; Stemgent), Oct-4 (1/200; Millipore), and Nanog (1/600; Santa Cruz). These primary antibodies were visualized with Alexa 488-conjugated goat anti-rabbit IgG, Alexa 594-conjugated goat anti-rabbit IgG, or Alexa Fluor 488-conjugated goat anti-mouse IgG (Invitrogen). Nuclei were stained with DAPI. Fluorescence images were acquired using a Zeiss inverted LSM confocal microscope (Carl Zeiss).

### Picking iPSC colonies

When the colonies became visible to the naked eye, we started to pick them manually. We gently scratched the colonies using a 100-μl tip and transferred one colony to one well of the 24-well plates precoated with Matrigel and E8 medium. We typically picked 10–20 colonies for each donor.

### Teratoma formation assay and histological analysis

Human iPSCs were suspended at 1 × 10^8^ cells/ml in PBS, and 100 μl of the cell suspension (1 × 10^7^ cells) was injected subcutaneously into the dorsal flank of SCID mice (five mice per cell line). One month after the injection, tumors were surgically dissected from the mice. Teratomas were weighted, fixed in PBS containing 4% formaldehyde, and embedded in paraffin. Sections were stained with hematoxylin and eosin.

### Gene expression analysis of iPSCs

To assess their self-renewal propensities, we collected MNCs, iPSCs, and reprogrammed cells at 4, 5, and 7 days and extracted total RNA using the RNeasy plus kit (Qiagen). Real-time PCR was performed using the SYBR Green PCR Master Mix (Applied Biosystems, Foster City, CA, USA) on a 7500 Fast Real-Time PCR System (Applied Biosystems). The primer sets were as follows: *Oct4*—Hoct4 FP, 5′-ATTCAGCCAAACGACCATCT-3′ and Hoct4 RP, 5′-GCTTCCTCCACCCACTTCT-3′; *Sox2*—HSox2 FP, 5′-CACACTGCCCCTCTCACACA-3′ and HSox2 RP, 5′-CCCTCCCATTTCCCTCGTTT-3′; and *Nanog*—HNanog FP, 5′-GCCGAAGAATAGCAATGGTGTG-3′ and HNanog RP, 5′-GGAAGATAGAGGCTGGGGTAG-3′. To determine the average copy numbers of residual or integrated episomal vector in iPSC clones, real-time PCR analysis was performed. Total DNA (genomic and episomal) was extracted from iPSCs at passage 10. Two sets of primers were used to detect episomal vector DNA (in either episomal or integrated form): EBNA1-F, 5′-TTTAATACGATTGAGGGCGTCT-3′ and EBNA1-R, 5′-GGTTTTGAAGGATGCGATTAAG-3′; and OSW-F, 5′-GGATTACAAGG ATGACGACGA-3′ and OSW-R, 5′-AAGCCATACGGGAAGCAATA-3′. The amplification program consisted of 50 °C for 2 min and 95 °C for 10 min, followed by 40 cycles at 95 °C for 15 s and 60 °C for 1 min.

### Karyotyping and G-banding

G-banding chromosome analysis of the iPSC line was performed following the protocol published by Li et al. [19]. Data were interpreted by a certified cytogenetic technologist.

### Propidium iodide staining of live/dead cells

We used a flow cytometry assay to determine the ratio of live/dead cells. Briefly, harvested cells were washed with PBS, and then the cell pellet was suspended in PBS with 1 μg/ml propidium iodide and samples maintained in that solution at 4 °C protected from light before analysis on a flow cytometer.

### Statistical analysis

Data are presented as mean ± SEM. Two-tailed Student *t* tests were performed, and *P* < 0.05 was considered statistically significant.

## Results

### Isolating MNCs from human peripheral blood by different methods

**Fig. 1:**
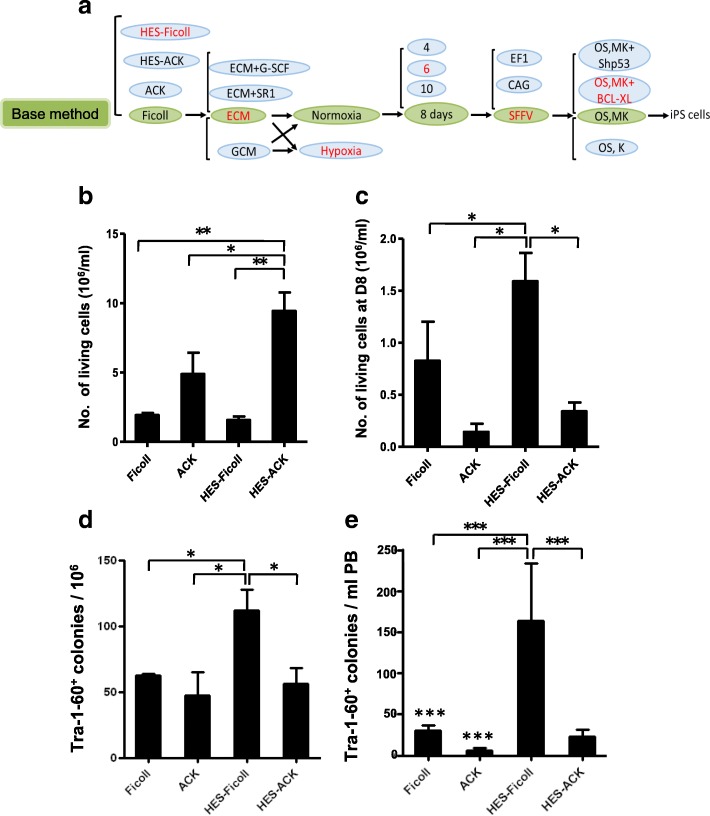
Different effects of four MNC isolation methods on reprogramming of PB MNCs with episomal vectors. **a** Flow chart of optimized method for generation of integration-free iPSCs from human PB. Green ellipses highlight basic method. For figuring out best conditions, one factor was optimized while controlling others following indicated basic method. For each condition identified, at least three donors randomly selected and repeated three times per donor. **b** Number of living MNCs isolated from 1 ml of PB by four methods at day 0. **c** Number of living MNCs isolated from 1 ml of PB after 8 days in culture. **d** Number of TRA-1-60-positive colonies generated from 1 × 10^6^ PB MNCs. **e** Number of TRA-1-60-positive colonies generated from 1 ml of PB by different isolating methods. PB MNCs cultured for 8 days before nucleofection. PB MNCs (1 × 10^6^ cells) nucleofected and then seeded into each well. TRA-1-60 staining of iPSCs at 3 weeks after nucleofection of PB MNCs with episomal vectors. Data presented as mean ± SEM (*n* = 3). *P < 0.05; **P < 0.01; ***P < 0.001. OS, pEV SFFV-OCT4-E2A-SOX2; MK, pEV SFFV-MYC-E2A-KLF4; Shp53, pEV SFFV-Shp53;BCL-XL, pEV SFFV-BCL-XL; K, pEV SFFV- KLF4. HES hydroxyethyl starch, ECM erythroid culture medium, GCM granulocyte culture medium, G-CSF granulocyte-colony stimulating factor, SR1 StemRegenin1, iPS induced pluripotent stem, D day, PB peripheral blood

PB MNCs are ideal for reprogramming iPSCs and have the potential to expedite advances in iPSC-based therapies [20]. To improve the generation efficiency of integration-free iPSCs from human PB MNCs, we optimized the method of generating them from human PB (Fig. 1a). In the first step, we used two isolating methods for enrichment of PB MNCs, Ficoll and HES-Ficoll, and two isolating methods without enrichment, ACK and HES-ACK. The total number of cells isolated from 1 ml of PB with the four methods changed significantly. Yields with ACK and HES-ACK were significantly greater than with Ficoll or HES-Ficoll (Fig. 1b, Additional file 1: Figure S1A). In theory, the number of MNCs per milliliter of blood was almost the same in different groups from the same donor. Nonetheless, after 8 days of in-vitro culture, the number of live cells in the HES-Ficoll group was significantly greater than in the other groups (Fig. 1c, Additional file 1: Figure S1A). This result suggested that the HES-Ficoll group that yielded relatively purified MNCs initially was beneficial to cell culture.

We then generated the iPSCs from these cultured MNCs with a combination of reprogramming factors consisting of OCT4, SOX2, KLF4, and C-MYC. The ESC-like and TRA-1-60-positive colonies began to emerge at 7 days after nucleofection. Compared with the other three groups, the HES-Ficoll group generated significantly more TRA-1-60-positive colonies in every 1 × 10^6^ live cells or every 1 ml of PB than the other three groups (Fig. 1d, e). This finding reveals that the different MNC isolation methods can affect iPSC generation.

### The effect of culture medium and culture time on the generation of integration-free iPSCs

**Fig. 2:**
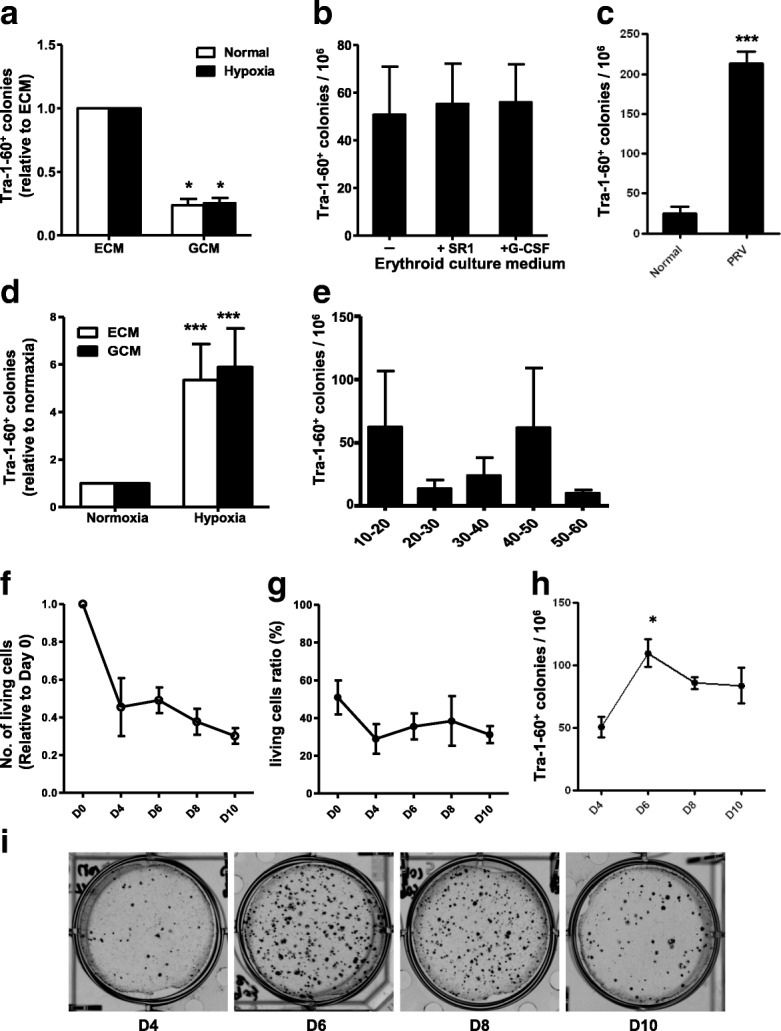
Optimized culture conditions for generation of integration-free iPSCs. **a** ECM can improve reprogramming in both normoxic and hypoxic conditions. **b** Adding StemRegenin1 (SR1) or granulocyte-colony stimulating factor (G-CSF) to ECM does not affect reprogramming. PB MNCs cultured for 8 days before nucleofection with episomal vectors expressing OS, MK. PB MNCs (1 × 10^6^ cells) nucleofected and then seeded into each well. Numbers of TRA-1-60-positive iPSC colonies counted 3 weeks after nucleofection. **c** Reprogramming efficiency of PB MNCs from four healthy volunteers and two polycythemia patients. **d** Hypoxia (3%) increases reprogramming efficiency under both ECM and GCM conditions. **e** Reprogramming efficiency of all healthy volunteers at different ages. PB MNCs cultured for 8 days before nucleofection with episomal vectors expressing OS, MK. PB MNCs (1 × 10^6^ cells) nucleofected and then seeded into each well. Numbers of TRA-1-60-positive iPSC colonies counted 3 weeks after nucleofection. **f** Number of living MNCs decreased after 10 days of culture with ECM. **g** Ratio of living MNCs changed after 10 days of culture with ECM. **h** Culturing PB MNCs for different numbers of days affected reprogramming efficiency. PB MNCs cultured for 4–10 days before nucleofection with episomal vectors expressing OS, MK. PB MNCs (1 × 10^6^ cells) nucleofected and then seeded into each well. Numbers of TRA-1-60-positive iPSC colonies counted 3 weeks after nucleofection. **i** AP staining photographs after different days of culture with SFFV promoter episomal vectors. Data representative of three experiments (mean ± SEM). *P < 0.05; ***P < 0.001. ECM erythroid culture medium, GCM granulocyte culture medium, PRV polycythemia vera, D day

CD34^+^ cells in PB MNCs would be expended and differentiated after culture in vitro. Different culture conditions can affect the efficiency of generating integration-free iPSCs from human PB [21]. ECM was used to expand and culture the PB MNCs and improve their reprogramming efficiency (Fig. 2a). Compared with ECM medium alone, adding StemRegenin1 (SR1, the inhibitor of the aryl hydrocarbon receptor) or G-CSF to the ECM medium did not improve the reprogramming (Fig. 2b). This result indicated that the nucleated erythrocyte cells may be reprogrammable cells with high efficiency, except for CD34^+^ cells.

To confirm our hypothesis, we selected PB cells from patients with polycythemia vera (PRV) disease, which is an uncommon neoplasm in which the bone marrow makes too many red blood cells. PRV involves elevated hemoglobin level and hematocrit, reflecting the increased number of circulating red blood cells. The results showed that the reprogramming efficiency of MNCs from a PRV patient’s PB was significantly increased (Fig. 2c). After nucleofection, PB MNCs were cultured under normoxia or hypoxic conditions, respectively. Three weeks later, the number of TRA-1-60-positive colonies formed under hypoxic conditions (3%) was four to six times higher than that in normoxic conditions (Fig. 2d).

To identify the effect of age on reprogramming, we selected healthy volunteers of different ages (10–20 years, five donors; 20–30 years, five donors; 30–40 years, four donors; 40–50 years, three donors; 50–60 years, three donors). The reprogramming efficiency was not affected by the age of the donors (Fig. 2e).

In addition to change the culture conditions, separated PB MNCs were expanded in vitro for 8–10 days, as reported previously [17]. With the longer culture time, the total number of living cells gradually decreased (Fig. 2f, Additional file 1: Figure S1B), while the percentage of living cells remained unchanged (Fig. 2g, Additional file 1: Figure S1B). This result indicated that there was a certain rate of cells dead every day. To confirm the optimal culture time, we transfected the cells that were cultured respectively at days 4, 6, 8, and 10. At 3 weeks after nucleofection, the number of TRA-1-60-positive colonies was greatest at day 6 (Fig. 2h), and this result was confirmed by the AP staining method (Fig. 2i).

### The effect of vectors on the generation of iPSCs from peripheral blood

**Fig. 3:**
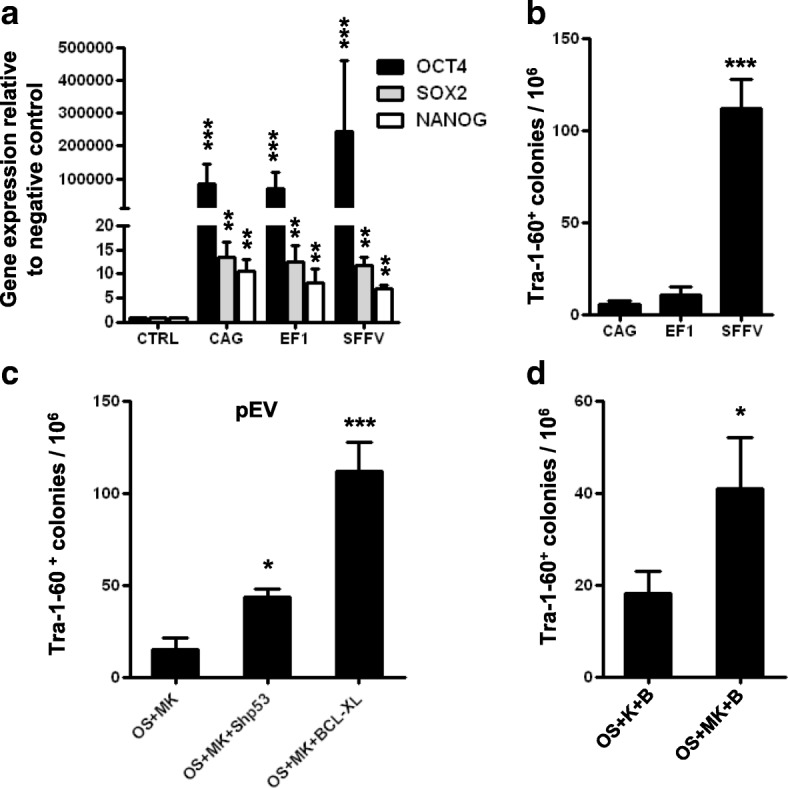
Generation of integration-free iPSCs from PB MNCs with different episomal vectors. **a** Expression level of pluripotent genes 48 h after nucleofection by real-time PCR. CTRL group, PB MNCs before nucleofection. **b** Number of TRA-1-60-positive colonies generated from 1 × 10^6^ PB MNCs 3 weeks after nucleofection. PB MNCs cultured for 8 days before nucleofection with episomal vectors (OS, MK) of different promoters (CAG, EF1, SFFV). PB MNCs (1 × 10^6^ cells) nucleofected and then seeded into each well. **c** Number of TRA-1-60-positive colonies generated from 1 × 10^6^ PB MNCs 3 weeks after nucleofection with different reprogramming factor-expressing episomal vectors. OS, pEV SFFV-OCT4-E2A-SOX2; MK, pEV SFFV-MYC-E2A-KLF4; Bcl-XL, pEV SFFV-BCL-XL; Shp53, pEV SFFV-Shp53. **d** Effect of c-MYC on generation of integration-free iPSCs from human PB. B, pEV SFFV-BCL2; K, pEV SFFV-KLF4. Data presented as mean ± SEM (*n* = 6) *P < 0.05, **P < 0.01, ***P < 0.001

We have noted that the reprogramming efficiency varied with different combinations of episomal vectors (pEB (C5 + Tg), pEV (OS+MK)), which may be associated with different promoters of these vectors [22]. MNCs from human PB were transfected with the pEV episomal vectors CAG, EF1, or SFFV, which had different promoters. At 48 h, the expression of pluripotent genes (Fig. 3a) did not differ among the different promoters. Three weeks later, the number of TRA-1-60-positive colonies was assessed, and the SFFV promoter looked propitious for the reprogramming of the human PB cells (Fig. 3b). We then compared the combination of transcription vectors and found that the different combinations of episomal vectors and transcription factors had different effects on the formation of iPSC colonies. OSMK and BCL-XL represented the best or the more efficient combination [18, 22] (Fig. 3c, d).

### Characterization of iPSC colonies generated from the human peripheral blood cells

**Fig. 4:**
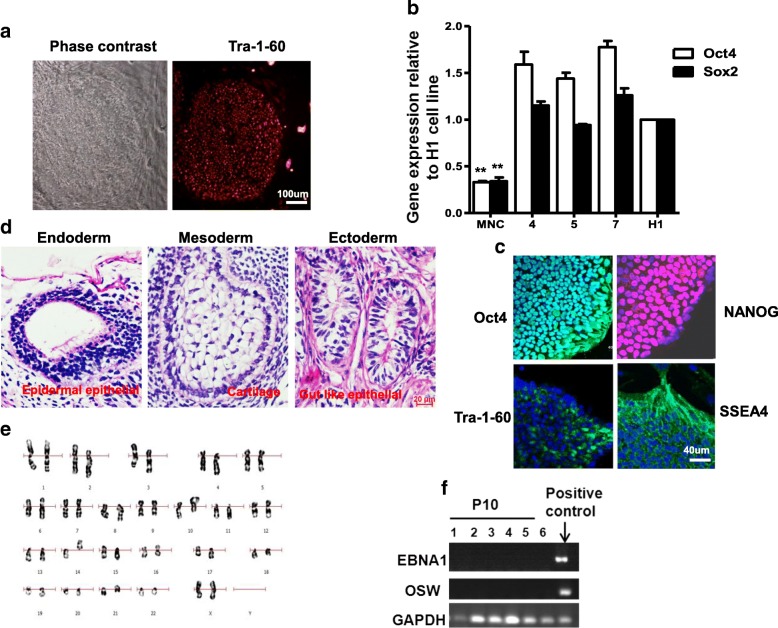
Characterization of integration-free iPSCs from PB MNCs. **a** Representative TRA-1-60 staining photograph of integration-free iPSC colony from PB MNCs. **b** Expression level of pluripotency genes of iPSCs compared with H1 by real-time PCR. **c** PB iPSCs expressed pluripotency markers OCT4, NANOG, TRA-1-60, and SSEA4. Representative images captured using Leica confocal microscope. **d** PB iPSCs formed teratoma in immunodeficient mice. H&E staining of representative teratoma from PB iPSCs with derivatives of three embryonic germ layers: cartilage (mesoderm), glands (endoderm), and neurotubules (ectoderm). **e** Representative karyotype of iPSC clone. All analyzed PB iPSC clones showed normal karyotype. **f** Vector sequence (EBNA1 and OSW) not found based on PCR-based detection in expanded iPSCs after 10 passages. MNC mononuclear cell, P passage

We have established that iPSCs generated from PB MNCs using the optimized methods (Table 1) are indistinguishable in their behavior in culture and colony morphology from those of ESCs (Fig. 4a). Three iPSC lines were picked from the PB-iPSCs, and the expression of the pluripotency genes *Oct4* and *Sox2* in these three iPSCs were coincident with the H1 ESCs by real-time PCR (Fig. 4b). By immunostaining assay, we found that clones of iPSCs established from human PB retained typical characteristics of pluripotent stem cells such as the expression of embryonic stem cell markers (e.g., Oct4, NANOG, TRA-1-60, and SSEA4) (Fig. 4c). PB-iPSCs could form teratomas and differentiate into the three embryonic germ layers in immunodeficient mice (Fig. 4d). Cytogenetic analysis of all PB iPSC colonies showed a normal karyotype (Fig. 4e). All of these data demonstrated the pluripotency of these iPSCs. Ultimately, according to previous reports [23, 24], we passaged the iPSCs beyond 10 passages, and PCR-based detection of the vector sequence (EBNA1 and OSW) was not found in the expanded iPSCs after 10 passages (Fig. 4f). When we established iPSC lines, we also observed a certain proportion of clones undergoing differentiation (Additional file 2: Figure S2) and death in the same well derived from the same PB sample, which may indicate that there are differences between the different clones obtained from the same PB sample using the same method of reprogramming and cultivation.

**Table 1:** Human iPSCs generated from PB with the optimized protocol

Sample number	Genetic background	Age (years)	Male/female	Efficiency (%)	Number of iPSC lines^a^
PB-1	Normal	39	Female	0.0004	4
PB-2	Normal	26	Female	0.001	5
PB-3	Normal	34	Female	0.002	8
PB-4	Normal	45	Female	0.002	13
PB-5	Normal	23	Female	0.0002	3
PB-6	Normal	20	Male	0.00053	12
PB-7	Normal	26	Male	0.0007	2
PB-8	Normal	47	Male	0.00253	1
PB-9	Normal	11	Male	0.0006	5
PB-10	Normal	59	Male	0.0009	3
PB-11	Normal	36	Male	0.00657	5
PB-12	Normal	10	Male	0.00387	8
PB-13	Normal	3	Male	0.001	3
PB-14	Normal	4	Male	0.0006	5
PB-15	Normal	28	Female	0.002	2
PB-16	Normal	24	Female	0.001	3
PB-17	Normal	26	Female	0.005	5
PB-18	Normal	24	Male	0.009	10
PB-19	Normal	23	Female	0.002	4
PB-20	Normal	20	Male	0.001	3
PB-21	Normal	24	Female	0.0008	7
PB-22	PRV	22	Male	0.00193	9
PB-23	PRV	22	Male	0.00243	9
PB-24	JMML	3	Male	0.0006	1
PB-25	JMML	3.9	Male	0.0008	2
PB-26	JMML	5	Male	0.00114	10

*iPSC* induced pluripotent stem cell, *JMML* juvenile myelomonocytic leukemia, *PB* peripheral blood, *PRV* polycythemia vera

^a^iPSC lines listed were identified by ESC characterization. We did not include iPSC lines without identification in the analysis

## Discussion

In the present study, we optimized the episomal method to generate integration-free iPSCs from PB MNCs to iPSCs. First, we found that much purer MNCs can be obtained from 1 ml of PB using the HES-Ficoll method compared to the other three options. After 6 days of in vitro culture, the most iPSC clones were acquired after transfection. ACK lysis buffer was used for lysis of the red blood cells. During this process, the polymorphonuclear cells were left in the ACK and HES-ACK procedures, which are not useful for MNC culture. On the other hand, Ficoll could not completely separate MNCs from red blood cells, while with the combination of HES and Ficoll most of the red blood cells could be precipitated and removed. MNCs could then be separated from the remaining cells with the least damage to themselves.

CD34^+^ cells respond well to the cytokine cocktail and are reprogrammable with high efficiency [6, 25–27]. In our study, we found that the erythroid culture medium improved reprogramming efficiencies, favoring the expansion of erythroblasts instead of lymphocytes [17]. Therefore, adding granulocyte growth factors such as SR1 or G-CSF to ECM did not change the efficiencies, indicating that erythroblasts are the most important donor cell source except for CD34^+^ cells and can be reprogrammed with high efficiency.

MNCs from PRV patient PB cells had a high induction efficiency in forming iPSCs (Fig. 2c). The possible reason for this is that the erythroblasts are in specific epigenetic states that are more easily reprogrammed [23]. The reported PBMC reprogramming experiment recommends that PB MNCs are expanded over the course of 8–14 days in the culture medium [17]. We generated iPSCs from PB MNCs that had been cultured for different time periods and confirmed that the optimal culture time is on day 6, based on comparing the number of TRA-1-60-positive and AP-positive colonies formed.

The virus encapsulated with SFFV as a vector can transfect human hematopoietic cells more efficiently and be expressed for a long time [28]. In our data, the expression of transcriptional genes did not increase significantly in the SFFV group at 48 h after transfection compared to the other promoters. We suggest that the persistence of expression may be the key reason for the high efficiency of reprogramming. Our results show that when the promoter of the episomal vector is SFFV, the reprogramming efficiency is most optimal (Fig. 3b).

Thus far, many studies have proved that different combinations of transcription factors can be applied successfully to cell reprogramming [20, 26]. BCL-XL is well known for acting as an antiapoptotic protein [29], which is beneficial [30]; in addition, OSMK with BCL-XL has the most positive effect on the formation of iPSC colonies [22] (Fig. 3c, d).

Earlier studies have reported that hypoxia can improve survival of neural spine cells [31] and hematopoietic stem cells [32] and can inhibit the differentiation of ESCs [33]. Our study also confirmed that hypoxic conditions can improve the reprogramming efficiency of PB MNCs after nucleofection.

## Conclusions

In the present study, we sought to improve the episomal method for generating iPSCs from PB MNCs and to lay some foundation for individualized iPSCs for future clinical application. With this optimized protocol, we improved the generation efficiency of integration-free iPSCs from human peripheral blood mononuclear cells, and a valuable asset for banking patient-specific iPSCs has been established.

## Additional files

Additional file 1: **Figure S1.** FACS staining of live/dead cells. **A** Representative images of FACS staining of live/dead cells of PB MNCs by four PB MNC isolation methods at day 0 or after 8 days. **B** Representative images of FACS staining of live/dead cells of PB MNCs at indicated time points. PB MNCs isolated with Ficoll method. (PPTX 99 kb)

Additional file 2: **Figure S2.** Differentiated PB iPSC clones did not express pluripotency markers OCT4, NANOG, TRA-1-60, and SSEA4. Representative images captured using Leica confocal microscope. (PPTX 292 kb)
