# Supplementary figures and images for: Optimizing the method for generation of integration-free induced pluripotent stem cells from human peripheral blood
Source: Stem Cell Res Ther. 2018 Jun 15;9:163. doi: 10.1186/s13287-018-0908-z (PMC6002980; doi:10.1186/s13287-018-0908-z)

## Slide 1
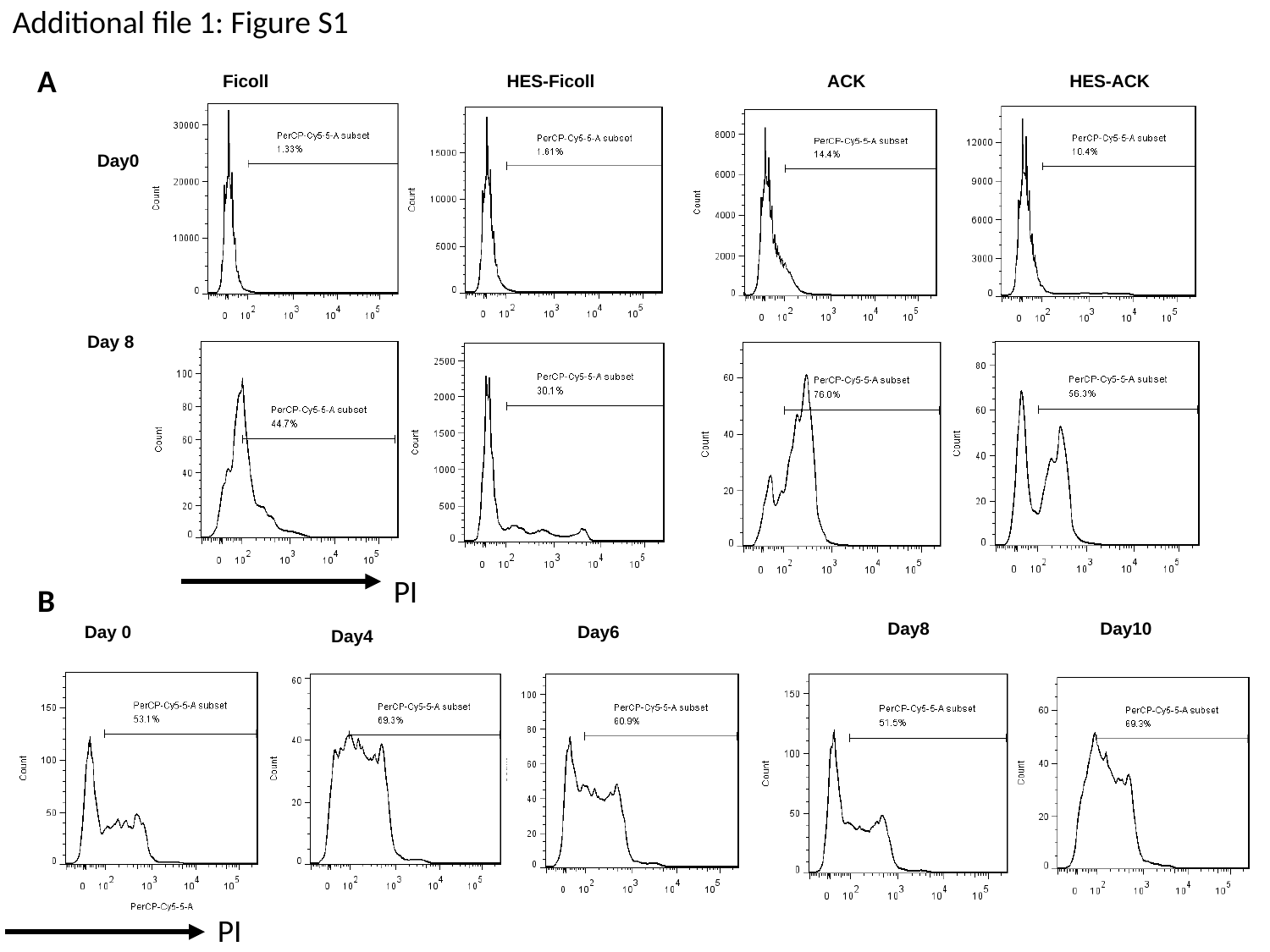

Additional file 1: Figure S1
A
Ficoll HES-Ficoll ACK HES-ACK
Day0
Day 8
PI
B
Day8
Day10
Day 0
Day6
Day4
PI

Supplement: Supplementary file 1 — Figure S1. FACS staining of live/dead cells. A Representative images of FACS staining of live/dead cells of PB MNCs by four PB MNC isolation methods at day 0 or after 8 days. B Representative images of FACS staining of live/dead cells of PB MNCs at indicated time points. PB MNCs isolated with Ficoll method. (PPTX 99 kb) [file 13287_2018_908_MOESM1_ESM.pptx]

## Slide 1
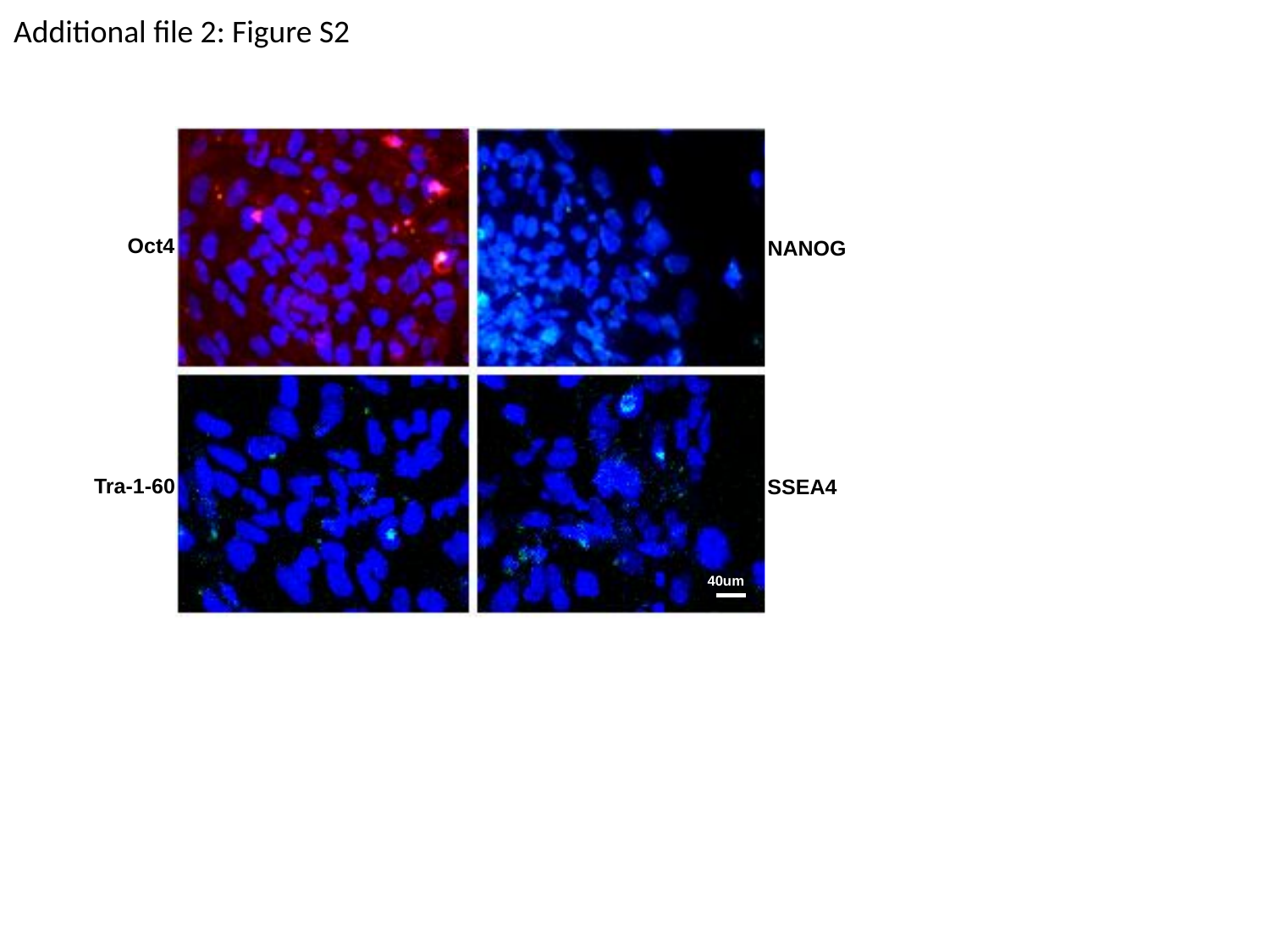

Additional file 2: Figure S2
Oct4
 NANOG
Tra-1-60
SSEA4
40um

Supplement: Supplementary file 2 — Figure S2. Differentiated PB iPSC clones did not express pluripotency markers OCT4, NANOG, TRA-1-60, and SSEA4. Representative images captured using Leica confocal microscope. (PPTX 292 kb) [file 13287_2018_908_MOESM2_ESM.pptx]
